# Differences in the epidemiology of out-of-hospital and in-hospital trauma deaths

**DOI:** 10.1371/journal.pone.0217158

**Published:** 2019-06-04

**Authors:** Ben Beck, Karen Smith, Eric Mercier, Belinda Gabbe, Richard Bassed, Biswadev Mitra, Warwick Teague, Josine Siedenburg, Susan McLellan, Peter Cameron

**Affiliations:** 1 Department of Epidemiology and Preventive Medicine, Monash University, Melbourne, Victoria, Australia; 2 Centre for Research and Evaluation, Ambulance Victoria, Doncaster, Victoria, Australia; 3 Department of Community Emergency Health and Paramedic Practice, Monash University, Frankston, Victoria, Australia; 4 Laval University, Quebec City, Quebec, Canada; 5 Emergency and Trauma Centre, The Alfred, Melbourne, Victoria, Australia; 6 Health Data Research UK, Swansea University Medical School, Swansea University, Swansea, United Kingdom; 7 Department of Forensic Medicine, Monash University, Melbourne, Victoria, Australia; 8 National Trauma Research Institute, Melbourne, Victoria, Australia; 9 Trauma Service, The Royal Children’s Hospital, Parkville, Victoria, Australia; 10 Department of Paediatrics, University of Melbourne, Parkville, Victoria, Australia; 11 Surgical Research Group, Murdoch Children’s Research Institute, Parkville, Victoria, Australia; University of Louvain, BELGIUM

## Abstract

**Background:**

Trauma is a leading cause of mortality. Holistic views of trauma systems consider injury as a public health problem that requires efforts in primary, secondary and tertiary prevention. However, the performance of trauma systems is commonly judged on the in-hospital mortality rate. Such a focus misses opportunities to consider all deaths within a population, to understand differences in in-hospital and out-of-hospital trauma deaths and to inform population-level injury prevention efforts. The aim of this study was to provide an epidemiological overview of out-of-hospital and in-hospital trauma deaths in a geographically-defined area over a 10-year period.

**Methods:**

We performed a population-based review of out-of-hospital and in-hospital trauma deaths over the period of 01 July 2006 to 30 June 2016 in Victoria, Australia, using data from the National Coronial Information System and the Victorian State Trauma Registry. Temporal trends in population-based incidence rates were evaluated.

**Results:**

Over the study period, there were 11,246 trauma deaths, of which 71% were out-of-hospital deaths. Out-of-hospital trauma deaths commonly resulted from intentional self-harm events (50%) and transport events (35%), while in-hospital trauma deaths commonly resulted from low falls (≤1 metre) (50%). The incidence of overall trauma deaths did not change over the study period (incidence rate ratio 0.998; 95%CI: 0.991, 1.004; P = 0.56).

**Conclusions:**

Out-of-hospital deaths accounted for most trauma deaths. Given the notable differences between out-of-hospital and in-hospital trauma deaths, monitoring of all trauma deaths is necessary to inform injury prevention activities and to reduce trauma mortality. The absence of a change in the incidence of both out-of-hospital and in-hospital trauma deaths demonstrates the need for enhanced activities across all aspects of injury prevention.

## Introduction

Trauma is a leading cause of mortality worldwide.[1, 2] Vital statistics systems are often used to record injury causes of death and changes over time.[1, 2] However, such systems do not enable the identification of the proportion of deaths that die in the out-of-hospital setting compared to the in-hospital setting.

Commonly, the performance of a trauma system is judged on the in-hospital mortality rate.[3, 4] However, such an approach fails to recognise the holistic view of trauma systems that contribute to reducing the burden of injury through primary, secondary and tertiary prevention efforts; that being contributions to injury prevention activities, prehospital care, in-hospital care, rehabilitation and community re-integration.[5] The focus of trauma systems on in-hospital mortality therefore misses opportunities to take a public health approach to injury and consider all trauma deaths within a population. This is reflected in numerous studies from trauma registries that rely solely on hospital data to inform injury prevention activities.[6–9] There is some evidence that out-of-hospital trauma deaths differ to in-hospital trauma deaths[10] and thus relying solely on hospital data may lead to missed opportunities for injury prevention.

Understanding differences in in-hospital and out-of-hospital trauma deaths is therefore vital in a comprehensive, coordinated and population-wide injury management system. The aim of this study was to provide an epidemiological overview of out-of-hospital and in-hospital trauma deaths in a geographically-defined area of Victoria, Australia over a 10-year period.

## Methods

### Study design

We performed a retrospective review of out-of-hospital and in-hospital trauma deaths over the period of 01 July 2006 to 30 June 2016 in Victoria, Australia using data from the National Coronial Information System (NCIS) and the Victorian State Trauma Registry (VSTR). Deaths of all ages were included in the study.

### Setting

The state of Victoria, Australia, has a population of 6.2 million people.[11] The Victorian State Trauma System is an inclusive, organised trauma system that was implemented between 2000 and 2003[4] with three hospitals (two adult, one paediatric) designated as major trauma services. A single ambulance service provides road and air (fixed wing and helicopter) transport of patients. Paramedics are authorised to withhold or cease resuscitation in the field as guided by Ambulance Victoria clinical practice guidelines when there is clear evidence of prolonged cardiac arrest or when injuries are incompatible with life; these have been described previously in more detail.[12]

### Data sources

#### Victorian state trauma registry

The VSTR is a population-based trauma registry that collects data about all hospitalised major trauma patients in the state of Victoria.[13] The registry includes data on the patient’s hospital admission including demographic, injury event, injury diagnosis, treatment and in-hospital outcomes. All patients that die in-hospital following injury, excluding patients with an isolated neck of femur fracture, are included on the registry.

In addition, the VSTR collects data on all trauma deaths in the state of Victoria through manual review of data from the NCIS. The VSTR and NCIS deaths were cross-checked to avoid double counting of deaths. Out-of-hospital trauma deaths were identified as those occurring prior to arrival at hospital.

#### National coronial information system

All deaths directly or indirectly resulting from injury or unnatural causes are reported to the State’s coroner. The NCIS is an Internet-based data storage and retrieval system for Australian coronial cases (http://www.ncis.org.au) and includes every death reported to the coroner since 2000. The NCIS contains coded data on the injury event, including the intent, mechanism of injury, trauma type and event location. In addition to these coded data fields, the NCIS contains full text documents, including the police report on the circumstances of the death, the autopsy report, and the forensic toxicology report. In this study, a small number of out-of-hospital trauma deaths were classified as ‘open coronial cases’ (n = 281; 2.6%) in which limited information was available on the intent and location of the event.

### Data analysis

Patient age was categorised into five groups: 0–4 years, 5–15 years, 16–34 years, 35–64 years and 65 plus years, reflecting age cut-offs that are relevant to the definitions, organisation and clinical practices of the Victorian State Trauma System.[14] Postcodes of the injury event were mapped to the Accessibility/Remoteness Index of Australia (ARIA) (a geographical index of remoteness), and postcodes of residence were mapped to the Index of Relative Socio-economic Advantage and Disadvantage (IRSAD) (which ranks areas in Australia according to relative socio-economic advantage and disadvantage). Trauma type was classified as blunt, penetrating, thermal mechanism (including contact with fire or flame and smoke inhalation), threat to breathing (including hangings, drownings, strangulation, other asphyxia and crush events) and other (including exposure to electrical current). A combination of NCIS data fields and International Statistical Classification of Diseases and Related Health Problems 10^th^ Revision–Australian Modification (ICD-10-AM) cause codes were used to classify the intent, trauma type and cause of injury. Where VSTR in-hospital deaths were not located on the NCIS, VSTR data fields were used to classify the intent, trauma type and cause of injury.

Data were summarised using percentages for categorical variables and median and interquartile range (IQR) for non-normally distributed continuous variables. Comparisons between age groups were conducted using χ^2^ or Kruskal-Wallis tests. Population-based incidence rates, and 95% confidence intervals (CI), were calculated for each year based on the total population at the start of each financial year (July 1 of the financial year to June 30 of the following year). Individual Poisson regression models were used to determine whether the incidence rate increased or decreased over the study period for all out-of-hospital trauma deaths, by trauma type, by intent, by mechanism of injury and by age group. Data were checked for potential over-dispersion (variance greater than the mean) to ensure that the assumptions of a Poisson distribution were met. The incidence rate ratio (IRR) and 95% CI were calculated. Deaths during the ‘Black Saturday’ bushfires that occurred in Victoria on the 7^th^ February 2009 (n = 180) were excluded from incidence calculations. A sensitivity analysis was conducted in which the ‘Black Saturday’ bushfire deaths were included, which is contained in the Supplementary Material. Data analysis was performed using Stata (Version 14.2, StataCorp, College Station, TX). A p-value <0.05 was considered statistically significant.

### Ethical approval

The VSTR has ethical approval from the Victorian Department of Health and Human Services HREC (DHHREC 11/14) and the Monash University HREC (CF13/3040–2001000165). The present study was approved by the Victorian Department of Justice and Regulation HREC (CF/16/272), and the Monash University HREC (CF16/532–2016000259).

## Results

Over the 10-year period, there were 11,246 trauma deaths, of which 8,032 (71%) were out-of-hospital deaths and 3,214 (29%) were in-hospital deaths. The overall crude incidence was 20.3 deaths per 100,000 population with an average of 1,125 trauma deaths per year. The crude incidence of out-of-hospital trauma deaths (14.4 deaths per 100,000 population) was greater than in-hospital trauma deaths (5.8 deaths per 100,000 population). Overall, these trauma deaths were mostly male (72%), occurred in major cities (64%), and resulted from unintentional (56%), and intentional self-harm (37%), events (Table 1). Transport events (32%) and hangings (24%) were the leading causes of injury.

**Table 1:** Demographic and injury data presented as overall, and for out-of-hospital and in-hospital trauma deaths. P-values reflect differences between out-of-hospital and in-hospital trauma deaths.

	Overall	Out-of-hospital deaths	In-hospital deaths	P-value
N	11246	8032	3214	
Age (years) ^a^				
0–4	123 (1.1%)	64 (0.8%)	59 (1.8%)	<0.001
5–15	224 (2.0%)	180 (2.2%)	44 (1.4%)	
16–34	3063 (27.2%)	2646 (33.0%)	417 (13.0%)	
35–64	4330 (38.5%)	3734 (46.5%)	596 (18.5%)	
65 years and older	3503 (31.2%)	1405 (17.5%)	2098 (65.3%)	
Sex ^b^				
Male	8120 (72.2%)	6143 (76.5%)	1977 (61.5%)	<0.001
Female	3123 (27.8%)	1886 (23.5%)	1237 (38.5%)	
IRSAD (quintiles) ^c^				
1^st^ (most disadvantaged)	1901 (17.7%)	1408 (18.5%)	493 (15.6%)	<0.001
2^nd^	1757 (16.3%)	1291 (17.0%)	466 (14.8%)	
3^rd^	2231 (20.7%)	1624 (21.4%)	607 (19.3%)	
4^th^	2406 (22.4%)	1706 (22.4%)	700 (22.2%)	
5^th^ (least disadvantaged)	2460 (22.9%)	1574 (20.7%)	886 (28.1%)	
ARIA ^d^				
Major Cities of Australia	6876 (63.6%)	4468 (58.5%)	2408 (76.0%)	<0.001
Inner Regional	3056 (28.3%)	2452 (32.1%)	604 (19.1%)	
Outer Regional or Remote Australia	871 (8.1%)	715 (9.4%)	156 (4.9%)	
Intent ^e^				
Unintentional	6105 (55.5%)	3348 (42.6%)	2757 (87.6%)	<0.001
Intentional Self-Harm	4118 (37.4%)	3926 (50.0%)	192 (6.1%)	
Assault	412 (3.7%)	312 (4.0%)	100 (3.2%)	
Other/Unknown	365 (3.3%)	265 (3.4%)	100 (3.2%)	
Trauma type ^f^				
Blunt	6249 (57.0%)	3497 (44.6%)	2752 (88.0%)	<0.001
Penetrating	788 (7.2%)	704 (9.0%)	84 (2.7%)	
Thermal Mechanism	408 (3.7%)	315 (4.0%)	93 (3.0%)	
Threat To Breathing	3425 (31.2%)	3252 (41.5%)	173 (5.5%)	
Other	95 (0.9%)	68 (0.9%)	27 (0.9%)	
Cause of injury				
Transport Injury Event	3568 (31.7%)	2786 (34.7%)	782 (24.3%)	<0.001
Low fall (≤1 m)	1671 (14.9%)	66 (0.8%)	1605 (49.9%)	
High fall (>1 m)	529 (4.7%)	283 (3.5%)	246 (7.7%)	
Other fall	178 (1.6%)	158 (2.0%)	20 (0.6%)	
Hanging	2669 (23.7%)	2568 (32.0%)	101 (3.1%)	
Other Crushing/Threat to Breathing	417 (3.7%)	363 (4.5%)	54 (1.7%)	
Contact with Person	73 (0.6%)	32 (0.4%)	41 (1.3%)	
Penetrating injury	774 (6.9%)	696 (8.7%)	78 (2.4%)	
Thermal mechanism	413 (3.7%)	315 (3.9%)	98 (3.0%)	
Drowning	421 (3.7%)	377 (4.7%)	44 (1.4%)	
Other/unknown	533 (4.7%)	388 (4.8%)	145 (4.5%)	

Missing data:

a) n = 3 (0.03%)

b) n = 3 (0.03%)

c) n = 491 (4.4%)

d) n = 443 (3.9%)

e) n = 246 (2.2%)

f) n = 281 (2.5%).

### Differences between out-of-hospital and in-hospital trauma deaths

**Fig 1:**
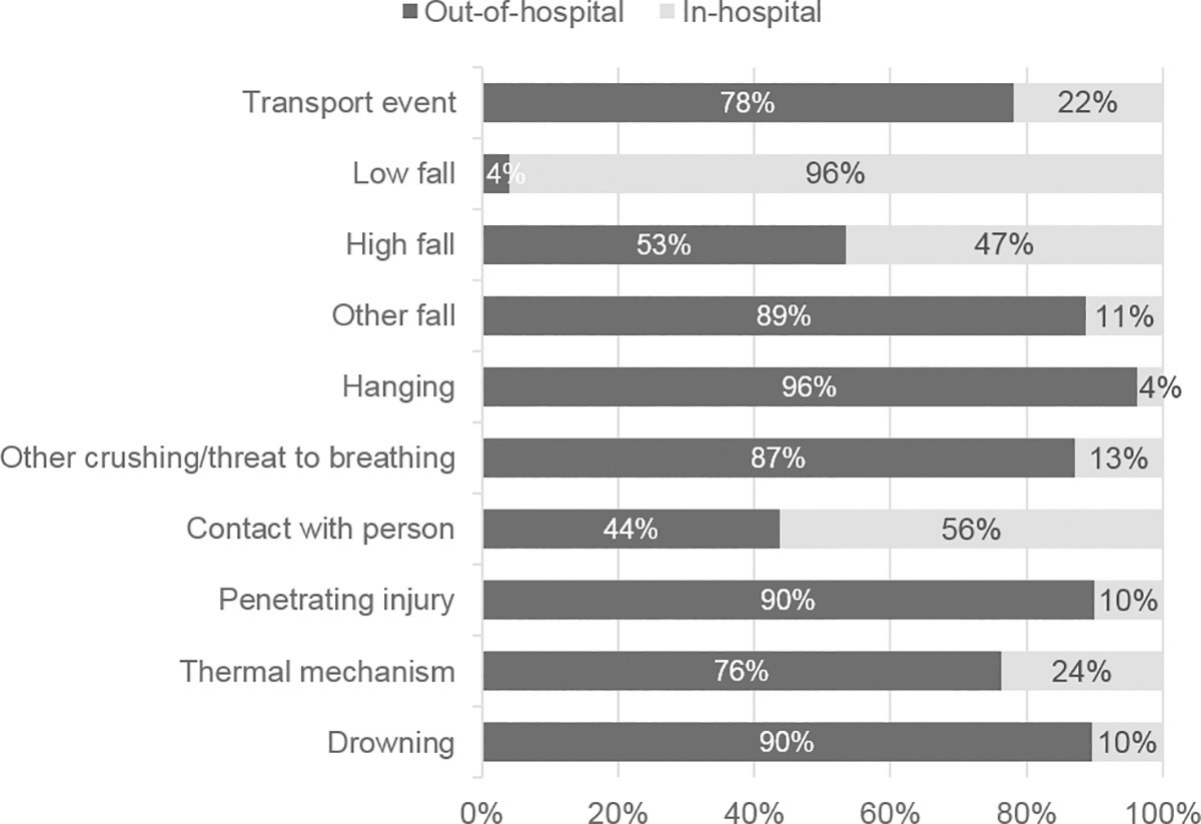
Proportional differences in out-of-hospital and in-hospital deaths by cause of injury.

Out-of-hospital trauma deaths more frequently occurred in younger age groups, with 18% of out-of-hospital trauma deaths occurring in people aged 65 years and older, compared to 65% of in-hospital trauma deaths (Table 1). A greater proportion of out-of-hospital trauma deaths occurred in inner and outer regional areas compared to in-hospital trauma deaths. Eighty-eight percent of in-hospital trauma deaths resulted from unintentional events, which were most commonly low falls. In contrast, 50% of out-of-hospital trauma deaths resulted from intentional self-harm events, which were most commonly hangings (Table 1). The following injury causes had the highest proportion of deaths in the out-of-hospital setting: transport events (78%; n = 2,786 of 3,568), hangings (96%; n = 2,568 of 2,669), penetrating injury (90%; n = 696 of 774) and drownings (90%; n = 377 of 421) (Fig 1). In contrast, the highest proportion of deaths following low falls were in the in-hospital setting (96%; n = 1,605 of 1,671) (Fig 1). Among the 7,740 deaths that occurred in people less than 65 years of age, only 14% (n = 1,116) were in-hospital deaths. Sex differences in the demographic and injury event characteristics are contained in the Supplementary material.

### Trends over time

**Fig 2:**
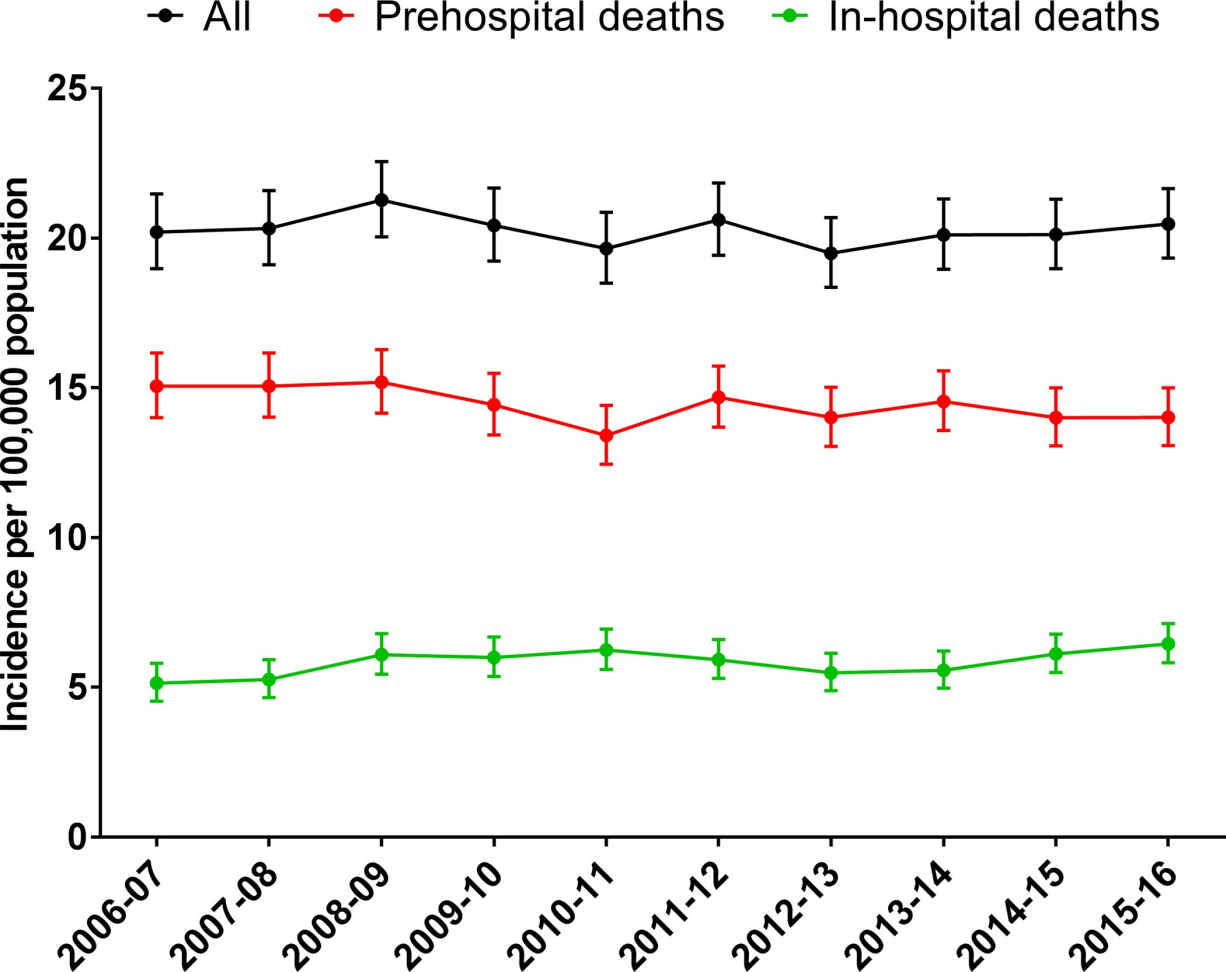
Incidence of out-of-hospital and in-hospital trauma deaths.

The incidence of overall trauma deaths did not change between July 2006 and June 2016 (IRR = 0.998; 95%CI: 0.991, 1.004; P = 0.56). In all ages, the incidence of out-of-hospital trauma deaths declined 0.8% per year (IRR = 0.992; 95% CI: 0.984, 0.999; P = 0.04) while the incidence of in-hospital trauma deaths increased 1.3% per year (IRR = 1.013; 95% CI: 1.002, 1.026; P = 0.03) (Fig 2). These findings were consistent in sensitivity analyses that included ‘Black Saturday’ bushfire deaths (Supplementary Material).

**Fig 3:**
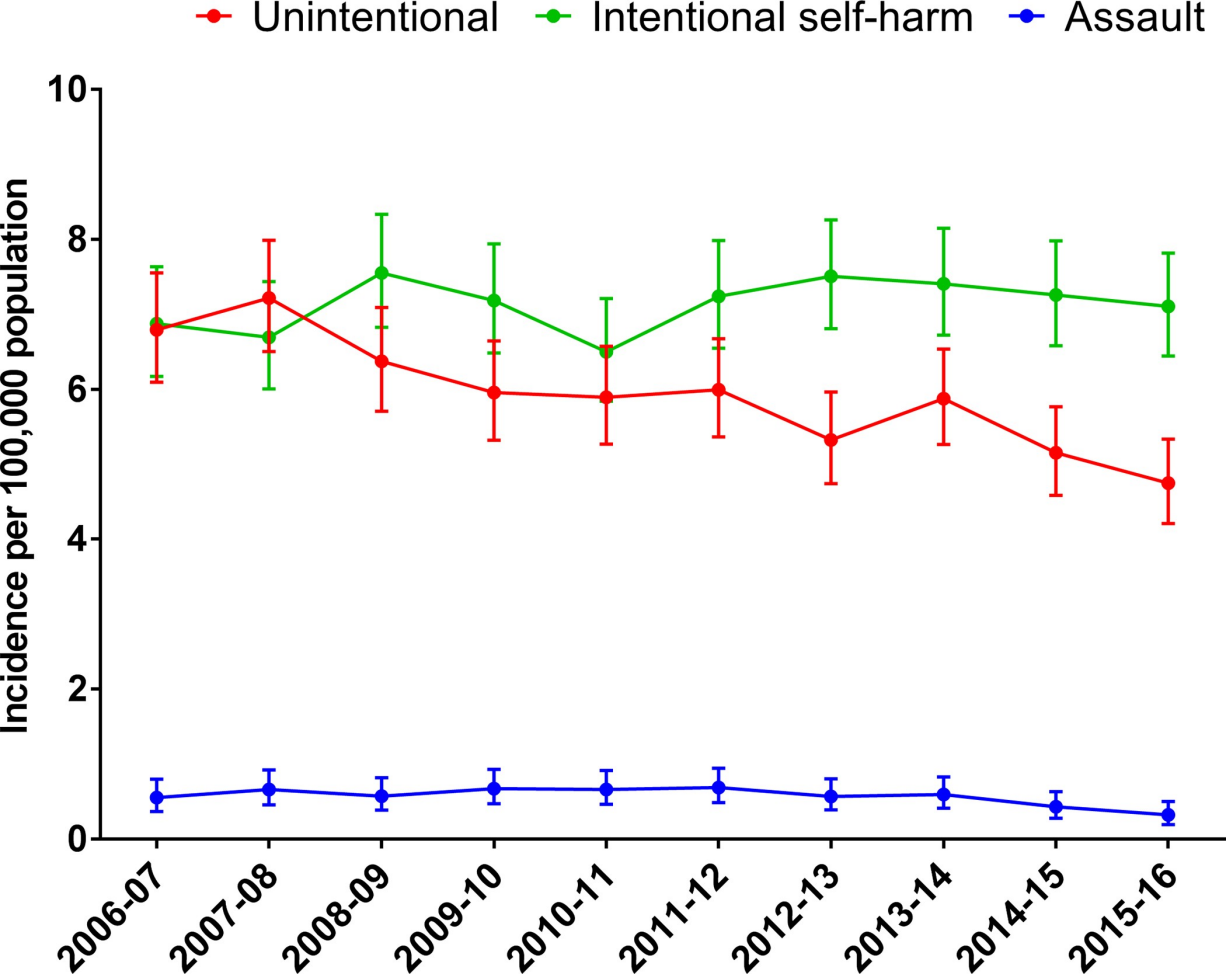
Incidence of out-of-hospital trauma deaths by the intent of the event.

Importantly, the size and direction of change in out-of-hospital trauma death rates was not consistent between age groups. The incidence of out-of-hospital trauma deaths declined 9% per year in people aged 5–15 years, 1.9% per year in people aged 16–34 years, but increased 2.4% per year in people aged 65 years and older (Table 2). Similarly, incidence trends varied with intent, such that of out-of-hospital trauma death resulting from unintentional events declined 3.7% per year, while the incidence of deaths resulting from intentional self-harm events did not change over the study period (Table 2 and Fig 3).

**Table 2:** Temporal trends in incidence over the period of 1 July 2006 to 30 June 2015.

	Incidence rate ratio (95% CI)	P-value	Incidence rate ratio (95% CI)	P-value
**ALL TRAUMA DEATHS**				
Overall	0.998 (0.991, 1.004)	0.560		
In-hospital	1.013 (1.002, 1.026)	0.027		
Out of hospital	0.992 (0.984, 0.999)	0.036		
	**OUT OF HOSPITAL TRAUMA DEATHS**		**IN-HOSPITAL TRAUMA DEATHS**	
***Age group***				
0–4 years	0.954 (0.875, 1.040)	0.284	0.967 (0.884, 1.058)	0.464
5–15 years	0.910 (0.863, 0.959)	<0.001	0.975 (0.880, 1.080)	0.629
16–34 years	0.981 (0.968, 0.994)	0.005	0.934 (0.903, 0.966)	<0.001
35–64 years	0.990 (0.979, 1.001)	0.086	0.981 (0.954, 1.009)	0.183
65 years and older	1.024 (1.005, 1.043)	0.012	1.029 (1.014, 1.044)	<0.001
***Sex***				
Males	0.990 (0.982, 0.999)	0.033	1.021 (1.004, 1.038)	0.016
Females	0.996 (0.981, 1.012)	0.658	1.038 (1.016, 1.061)	0.001
***Intent***				
Unintentional	0.963 (0.951, 0.974)	<0.001	1.015 (1.002, 1.028)	0.027
Intentional self-harm	1.006 (0.995, 1.017)	0.294	1.021 (0.972, 1.073)	0.404
Assault	0.957 (0.921, 0.995)	0.027	0.892 (0.832, 0.957)	0.001
***Trauma type***				
Blunt	0.965 (0.954, 0.759)	<0.001	1.011 (0.998, 1.024)	0.097
Penetrating	0.980 (0.955, 1.005)	0.124	0.955 (0.886, 1.029)	0.222
Thermal	0.949 (0.907, 0.995)	0.031	0.973 (0.905, 1.046)	0.451
Threat to breathing	1.010 (0.998, 1.022)	0.096	1.043 (0.990, 1.099)	0.116
***Mechanism of injury***				
Transport event	0.957 (0.945, 0.970)	<0.001	0.957 (0.933, 0.981)	0.001
Fall (any)	1.008 (0.978, 1.039)	0.621	1.073 (1.053, 1.094)	<0.001
Hanging	1.009 (0.996, 1.023)	0.172	1.076 (1.002, 1.156)	0.043
Other crushing / threat to breathing	1.015 (0.980, 1.053)	0.402	1.014 (0.924, 1.113)	0.770
Contact with person	1.028 (0.911, 1.161)	0.650	0.896 (0.802, 1.000)	0.051
Penetrating: stabbing	0.977 (0.930, 1.026)	0.346	0.871 (0.776, 0.976)	0.018
Penetrating: Firearm	0.967 (0.934, 1.001)	0.055	1.000 (0.894, 1.119)	0.994
Penetrating: Other	1.018 (0.953, 1.087)	0.601	1.327 (0.819, 2.149)	0.250
Thermal	0.950 (0.907, 0.995)	0.031	0.973 (0.905, 1.046)	0.451
Drowning	1.001 (0.966, 1.036)	0.974	0.983 (0.877, 1.101)	0.762
***Transport-specific***				
Motor vehicles	0.945 (0.929, 0.961)	<0.001	0.968 (0.934, 1.003)	0.070
Motorcycles	0.955 (0.923, 0.989)	0.010	0.936 (0.867, 1.010)	0.088
Pedestrian	0.982 (0.957, 1.008)	0.180	0.940 (0.899, 0.982)	0.006
Pedal cyclist	0.977 (0.889, 1.073)	0.626	0.965 (0.862, 1.080)	0.534
***Falls-specific***				
Fall <1m	1.077 (0.988, 1.172)	0.092	1.139 (1.110, 1.180)	<0.001
Fall ≥1m	0.966 (0.928, 1.006)	0.095	0.901 (0.855, 0.949)	<0.001
Stairs / steps	1.172 (1.015, 1.354)	0.031	1.079 (1.012, 1.151)	0.020

**Fig 4:**
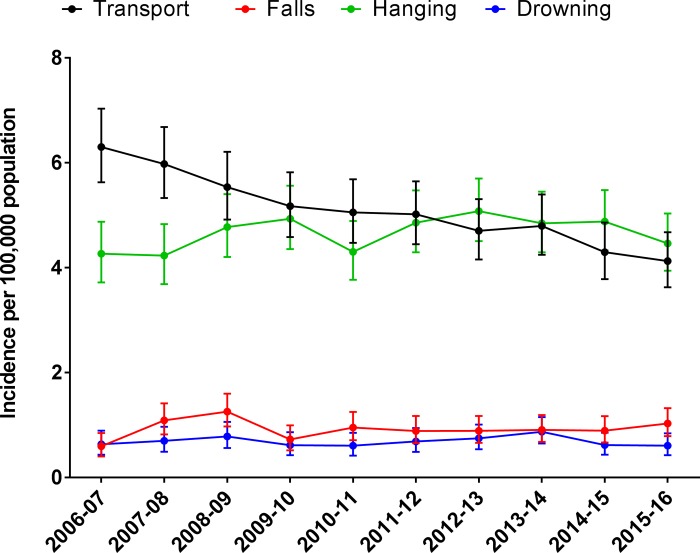
The incidence of out-of-hospital trauma deaths by mechanism of injury.

Out-of-hospital deaths from transport events declined 4.3% per year, which was largely driven by declines in motor vehicle occupant deaths (Table 2), while the incidence of out-of-hospital deaths resulting from hangings did not change over the study period (Fig 4). As a result, the incidence of hangings in the 2015/16 financial year (4.5 per 100,000 population) was greater than transport events (4.1 per 100,000 population).

The overall increase in the incidence of in-hospital trauma deaths was largely explained by increases in those aged 65 years and older, which increased 2.9% per year, and events resulting from low falls, which increased 13.9% per year (Table 2).

Demographic and injury event differences between age groups are contained in Table 3.

**Table 3:** Demographic and injury event profile of cases by age group for out-of-hospital trauma deaths.

	0–4 years	5–15 years	16–34 years	35–64 years	65 plus years	P-value
Number of out-of-hospital trauma deaths	48 (0.9%)	136 (2.4%)	1,858 (32.8%)	2,643 (46.7%)	976 (17.2%)	
*Demographics*						
Sex ^a^						<0.001
Male	28 (58.3%)	90 (66.2%)	1,446 (77.9%)	2,063 (78.1%)	683 (70.0%)	
Female	20 (41.7%)	46 (33.8%)	411 (22.1%)	578 (21.9%)	293 (30.0%)	
IRSAD (quintiles) ^b^ *						0.104
1^st^ (most disadvantaged)	7 (17.5%)	22 (17.7%)	328 (18.7%)	461 (18.5%)	170 (18.6%)	
2^nd^	10 (25.0%)	30 (24.2%)	289 (16.5%)	418 (16.8%)	161 (17.6%)	
3^rd^	9 (22.5%)	24 (19.4%)	378 (21.6%)	545 (21.9%)	176 (19.3%)	
4^th^	12 (30.0%)	31 (25.0%)	402 (23.0%)	563 (22.6%)	190 (20.8%)	
5^th^ (least disadvantaged)	^^	17 (13.7%)	353 (20.2%)	504 (20.2%)	217 (23.7%)	
ARIA ^c^ **						<0.001
Major cities	15 (36.6%)	66 (53.7%)	1,101 (62.4%)	1,413 (56.3%)	538 (59.1%)	
Inner regional	22 (53.7%)	47 (38.2%)	502 (28.5%)	887 (35.3%)	280 (30.7%)	
Outer regional / remote	^^	10 (8.1%)	161 (9.1%)	211 (8.4%)	93 (10.2%)	
*Injury event*						
Intent ^d^						<0.001
Unintentional	41 (91.1%)	85 (63.9%)	782 (43.1%)	942 (36.5%)	502 (52.5%)	
Intentional self-harm	-	34 (25.6%)	894 (49.3%)	1,445 (55.9%)	399 (41.7%)	
Assault	^^	9 (6.8%)	69 (3.8%)	115 (4.5%)	24 (2.5%)	
Other / unknown	-	5 (3.8%)	70 (3.9%)	82 (3.2%)	31 (3.2%)	
Trauma type ^e^						<0.001
Blunt	22 (48.9%)	70 (52.6%)	900 (49.6%)	961 (37.2%)	436 (45.9%)	
Penetrating	^^	6 (4.5%)	110 (6.1%)	248 (9.6%)	121 (12.8%)	
Threat to breathing	16 (35.6%)	40 (30.1%)	740 (40.8%)	1,208 (46.8%)	327 (34.5%)	
Thermal mechanism	5 (11.1%)	16 (12.0%)	51 (2.8%)	136 (5.3%)	53 (5.6%)	
Other	-	^^	13 (0.7%)	29 (1.1%)	12 (1.3%)	

* Note: Postcodes of residence were mapped to the Index of Relative Socioeconomic Advantage and Disadvantage (IRSAD).

** Note: Postcodes of the injury event were mapped to the Accessibility / Remoteness Index of Australia (ARIA). Missing data:

a) n = 133

b) n = 474

c) n = 441

d) n = 257

e) n = 177.

^^ denotes cell counts <5.

### Out-of-hospital trauma deaths: Unintentional events

Of the 3,347 out-of-hospital trauma deaths resulting from unintentional events, most were male (73%), occurred in those aged 16–34 years (34%) and 35–64 years (40%), and the leading cause of injury was transport events (67%) (Table 4). Of all unintentional falls, 27% were falls from height, 21% low falls, 9% falls down stairs and 44% in which the fall event could not be further classified. Of the high falls, 29% were from a balcony, roof or window, 22% were from a ladder and 45% could not be further classified.

**Table 4:** Cause of injury and location of event of cases by age group for out-of-hospital deaths resulting from unintentional events.

Unintentional event	Overall	0–4 years	5–15 years	16–34 years	35–64 years	65 years and older	P-value
Number of out-of-hospital trauma deaths resulting from unintentional events	3,347	54 (1.6%)	117 (3.5%)	1123 (33.5%)	1325 (39.6%)	728 (21.8%)	
*Sex* ^*a*^							<0.001
Male	2455 (73.4%)	30 (55.6%)	81 (69.2%)	899 (80.1%)	1013 (76.5%)	432 (59.3%)	
Female	891 (26.6%)	24 (44.4%)	36 (30.8%)	223 (19.9%)	312 (23.5%)	296 (40.7%)	
*Mechanism of injury* ^*b*^							
Transport event	2239 (66.9%)	20 (37.7%)	79 (67.5%)	903 (80.6%)	883 (66.6%)	354 (48.8%)	<0.001
Fall (any)	297 (8.9%)	-	^^	19 (1.7%)	80 (6.0%)	197 (27.1%)	
Hanging	30 (1%)	^^	^^	8 (0.7%)	15 (1.1%)	^^	
Other Crushing/Threat to Breathing	134 (4.0%)	5 (9.4%)	^^	26 (2.3%)	69 (5.2%)	30 (4.1%)	
Penetrating: stabbing	^^	-	-	^^	-	-	
Penetrating: shot by firearm	14 (0.4%)	-	-	8 (0.7%)	5 (0.4%)	^^	
Penetrating: scratching, cutting, etc.	22 (0.7%)	-	-	^^	13 (1.0%)	5 (0.7%)	
Thermal mechanism	242 (7.2%)	7 (13.2%)	14 (12.0%)	54 (4.8%)	112 (8.5%)	55 (7.6%)	
Drowning	239 (7.1%)	17 (32.1%)	12 (10.3%)	71 (6.3%)	87 (6.6%)	52 (7.2%)	
Other	124 (3.7%)	^^	^^	27 (2.4%)	61 (4.6%)	30 (4.1%)	
*Location of event*							<0.001
Highway, street or road	2056 (61.4%)	13 (24.1%)	55 (47.0%)	879 (78.3%)	817 (61.7%)	292 (40.1%)	
Home	564 (16.9%)	30 (55.6%)	22 (18.8%)	71 (6.3%)	237 (17.9%)	204 (28.0%)	
Farm	134 (4.0%)	^^	14 (12.0%)	16 (1.4%)	57 (4.3%)	46 (6.3%)	
Industrial or construction area	58 (1.7%)	-	-	16 (1.4%)	38 (2.9%)	^^	
Other	535 (16.0%)	10 (18.5%)	26 (22.2%)	141 (12.6%)	176 (13.3%)	182 (25.0%)	

Missing data:

a) n = 1

b) n = 5.

The mechanism of injury category ‘other’ included crush events / threat to breathing (n = 132), penetrating mechanisms (n = 32), and exposure to electricity (n = 18).

^^ denotes cell counts <5.

### Out-of-hospital trauma deaths: Intentional self-harm events

Of the 3,926 out-of-hospital trauma deaths resulting from intentional self-harm, most were male (81%), aged between 35–64 years (52%), and the leading cause of injury was hangings (64%), followed by transport events (11%) (Table 5). Most intentional self-harm transport events were pedestrian impacts with trains (75%). There were 40 cases (9%) of intentional self-harm motor vehicle collisions with trees or poles. Of all intentional falls, 42% were from a balcony, roof or window, and 43% were from a bridge. Self-cutting events and drownings each accounted for 2% of intentional self-harm deaths. Of the 198 (5%) intentional self-harm events classified with a cause of ‘other crushing/threat to breathing’, 93% (n = 185) resulted from intentional asphyxiation from plastic bags.

**Table 5:** Cause of injury and location of event of cases by age group for out-of-hospital trauma deaths resulting from intentional self-harm events.

Intentional self-harm event	Overall	0–4 years	5–15 years	16–34 years	35–64 years	65 years and older	P-value
Number of out-of-hospital trauma deaths resulting from intentional self-harm events	3,925	0	45 (1.1%)	1267 (32.3%)	2041 (52%)	572 (14.6%)	
*Sex*							<0.001
Male	3165 (80.6%)	-	26 (57.8%)	997 (78.7%)	1661 (81.4%)	481 (84.1%)	
Female	760 (419.4%)	-	19 (42.2%)	270 (21.3%)	380 (18.6%)	91 (15.9%)	
*Mechanism of injury*							<0.001
Transport event	423 (10.8%)	-	8 (17.8%)	203 (16.0%)	175 (8.6%)	37 (6.5%)	
Fall (any)	187 (4.8%)	-	^^	85 (6.7%)	91 (4.5%)	9 (1.6%)	
Hanging	2519 (64.2%)	-	31 (68.9%)	856 (67.6%)	1373 (67.3%)	259 (45.3%)	
Other Crushing/Threat to Breathing	198 (5.0%)		^^	36 (2.8%)	85 (4.2%)	76 (13.3%)	
Penetrating: stabbing	39 (1.0%)		-	7 (0.6%)	24 (1.2%)	8 (1.4%)	
Penetrating: shot by firearm	311 (7.9%)		^^	50 (3.9%)	154 (7.5%)	104 (18.2%)	
Penetrating: scratching, cutting, etc.	77 (2.0%)		-	6 (0.5%)	49 (2.4%)	22 (3.8%)	
Thermal mechanism	49 (1.2%)		-	6 (0.5%)	34 (1.7%)	9 (1.6%)	
Drowning	90 (2.3%)	-	-	16 (1.3%)	43 (2.1%)	31 (5.4%)	
Other	32 (0.8%)	-	-	^^	13 (0.6%)	17 (3.0%)	
*Location of event*							<0.001
Highway, street or road	190 (4.8%)	-	^^	77 (6.1%)	97 (4.8%)	15 (2.6%)	
Home	2614 (66.6%)	-	30 (66.7%)	775 (61.2%)	1385 (67.9%)	424 (74.1%)	
Farm	53 (1.3%)	-	^^	14 (1.1%)	31 (1.5%)	7 (1.2%)	
Industrial or construction area	57 (1.5%)	-	^^	19 (1.5%)	35 (1.7%)	^^	
Other	1011 (25.8%)	-	12 (26.7%)	382 (30.1%)	493 (24.2%)	124 (21.7%)	

Note: The cause of injury category ‘other’ included crush events / threat to breathing (n = 142), contact with fire or flame (n = 33) and exposure to electricity (n = 11).

^^ denotes cell counts <5.

## Discussion

In this population-based study of out-of-hospital and in-hospital trauma deaths over a 10-year period, we demonstrated no change in the incidence of all trauma deaths. We observed age-related shifts in trauma death rates, with declines in children and increases in adults, particularly older adults. Intentional self-harm events accounted for half of all out-of-hospital trauma deaths and, in the most recent year of data, hangings were the leading cause of out-of-hospital trauma deaths.

Overall, 71% of trauma deaths occurred out-of-hospital. While regionalised trauma systems have been demonstrated to reduce in-hospital mortality,[4, 15, 16] in most trauma systems, surveillance of out-of-hospital trauma deaths has often been neglected. Our results demonstrate the importance of a more inclusive approach to trauma death surveillance which captures both out-of-hospital and in-hospital deaths.

Population-based studies of out-of-hospital trauma deaths are rare. The incidence of out-of-hospital trauma deaths observed in our study (14.4 deaths per 100,000 population) was higher than that previously reported in Scotland (5.7 per 100,000 population).[17] This may be explained by differences in inclusion criteria, as certain intentional self-harm events (hangings and asphyxiation) and drownings were excluded from the Scottish study. These events accounted for over 50% of our out-of-hospital trauma deaths.

We observed important differences between out-of-hospital and in-hospital trauma deaths. Most in-hospital trauma deaths resulted from unintentional events, predominantly low falls, while the majority of out-of-hospital trauma deaths resulted from intentional self-harm events, predominantly hangings. These findings are consistent with data from Sweden.[10] We have also previously noted a substantial age-related shift in hospitalised major trauma with an increasing proportion of older adults with injuries resulting from low falls.[14] The high in-hospital mortality rate of older patients who present following a low fall has been associated with high rates of pre-existing comorbidities and in-hospital complications.[18, 19]

Almost one in two out-of-hospital trauma deaths resulted from unintentional events, which is consistent with prior studies.[10, 20] While continual reductions in transport-related mortality have been observed since the introduction of the seat belt in the 1970’s,[21] the large proportion of trauma deaths that continue to result from transport events demonstrates the need for greater investment in road safety if we are to meet current road safety objectives of eliminating deaths from road trauma.[22–24] While we observed a small proportion of out-of-hospital trauma deaths resulting from high falls, which were a combination of falls from a balcony, roof or window, or a ladder, high falls are a common cause of hospitalised major trauma, and additional public health campaigns are warranted.[6, 25] Drownings represented 5% of out-of-hospital trauma deaths and the incidence did not change over the study period. Globally, drowning is a common cause of death among children,[26] and our findings support this. Closer supervision of children around water, education around risks, greater teaching of swimming and further training in resuscitation may reduce deaths from drowning.[26] Alcohol and drugs have been identified as significant risk factors for adult drowning fatalities[27, 28] and targeted public health campaigns may be required to address these issues.

Importantly, our results demonstrated that deaths resulting from intentional self-harm accounted for half of all out-of-hospital trauma deaths. Suicide has been identified as a leading cause of injury mortality globally[29, 30] and is the leading cause of death in Australians aged 15–44 years.[2] Our results demonstrated that hangings accounted for nearly two-thirds of these intentional self-harm events. This is consistent for suicides of all-causes in Australia, not just limited to trauma, where hangings account for more than half of all suicide deaths, followed by poisoning by drugs, which accounted for 14% of suicide deaths.[31] Hangings and suffocations as a mechanism of intentional-self harm have been reported to be on the rise in the United States of America[32] and this has been attributed to displacement by preventative efforts targeting other mechanisms, including motor vehicle exhaust.[33, 34] Prevention strategies focused around restriction of access to means of hanging are of limited value and it has been suggested that primary prevention of suicide is likely to be the most effective approach.[35] The majority of intentional self-harm deaths resulting from transport events occurred as a result of pedestrian impacts with trains. This has been recognised as an international problem, however limited evidence exists for effective prevention practices.[36]

As intentional self-harm events were the leading cause of out-of-hospital trauma deaths, it is evident that further efforts in primary prevention are warranted. In 2015, the Australian Government established a new National Suicide Prevention Strategy; a systems-based approach to suicide prevention.[37] This national approach should lead to enhanced primary prevention efforts, including means restriction, improved physician education on the diagnosis and treatment of depression, enhanced screening and gatekeeper education.[38]

A trauma system should aim to reduce the burden of injury through primary, secondary and tertiary prevention efforts.[5] This comprehensive public health approach to injury management therefore needs to include surveillance of all trauma deaths, not just those who survive to reach hospital, which is a common limitation of trauma registries. Noting that nearly three-quarters of the total number of trauma deaths occur out-of-hospital and the major differences in the characteristics of in-hospital and out-of-hospital deaths, our results highlight the importance of comprehensive surveillance systems that include all trauma deaths and have the ability to identify out-of-hospital and in-hospital deaths. This is essential to achieving, measuring and sustaining reductions in death and injury through appropriately targeted injury prevention measures.

The strengths of this study include the population-based capture of all causes of out-of-hospital and in-hospital trauma deaths. Furthermore, we utilised multiple NCIS data fields to characterise events resulting in out-of-hospital trauma deaths and were not solely reliant on ICD-10 causes of death codes. Relying solely on ICD-10 codes is a method that is commonly used in trauma deaths studies and has been shown to result in classification errors in up to 30% of cases.[39] However, this study is not without limitations. A small proportion of cases were ‘open’ coronial cases and thus had limited event information. Further, specific details about some causes of injury (e.g. 45% missing data on the type of high fall) were missing. Furthermore, post-discharge deaths were not included in this study. We were not able to quantify the proportion of deaths attended by emergency medical services. As a result, we were unable to quantify the timing of out-of-hospital deaths. Additionally, delays in accessing closed coronial cases limits the currency of these data. While the focus of this study was to investigate the epidemiology of in-hospital and out-of-hospital trauma deaths, reviewing out-of-hospital trauma deaths provides an opportunity to examine the entire system of care provided to trauma patients, not limited to those that survive to hospital. We have previously used an expert panel review methodology to identify opportunities to reduce trauma mortality,[40, 41] and recommend that all trauma systems consider utilising a similar methodology to improve systems of care.

## Conclusion

In this comprehensive and population-based review of out-of-hospital trauma deaths, almost three-quarters of trauma deaths occurred in the out-of-hospital setting. Intentional self-harm and transport events were the leading cause of injury for out-of-hospital trauma deaths, while low falls were the predominant cause of in-hospital trauma deaths. Overall incidence of trauma deaths demonstrated little change, highlighting the need for enhanced and data-driven injury prevention strategies.

## Supporting information

S1 Table Sex differences in the demographic and injury event characteristics of out-of-hospital trauma deaths. (DOCX)

S1 Fig Incidence of out-of-hospital and in-hospital trauma deaths (including Black Saturday bushfire deaths). (DOCX)
