# Supplementary material for: Differences in the epidemiology of out-of-hospital and in-hospital trauma deaths
Source: PLoS One. 2019 Jun 4;14(6):e0217158. doi: 10.1371/journal.pone.0217158 (PMC6548370; doi:10.1371/journal.pone.0217158)
Supplement: S1 Table — (DOCX) [file pone.0217158.s001.docx]

S1 Table: Sex differences in the demographic and injury event characteristics of out-of-hospital trauma deaths.

|  | **Males** | **Females** | **P-value** |
| --- | --- | --- | --- |
| Number of out-of-hospital trauma deaths | 6143 | 1886 |  |
| *Demographics* |  |  |  |
| Age (years) ^a^ |  |  |  |
| 0-4 | 36 (0.6%) | 28 (1.5%) | <0.001 |
| 5-15 | 118 (1.9%) | 62 (3.3%) |  |
| 16-34 | 2075 (33.8%) | 570 (30.2%) |  |
| 35-64 | 2931 (47.7%) | 802 (42.5%) |  |
| 65 years and older | 982 (16.0%) | 423 (22.4%) |  |
| IRSAD (quintiles) ^b^ * |  |  |  |
| 1^st^ (most disadvantaged) | 1110 (19.0%) | 298 (16.9%) | 0.052 |
| 2^nd^ | 1010 (17.3%) | 281 (15.9%) |  |
| 3^rd^ | 1248 (21.4%) | 376 (21.3%) |  |
| 4^th^ | 1284 (22.0%) | 422 (23.9%) |  |
| 5^th^ (least disadvantaged) | 1183 (20.3%) | 391 (22.1%) |  |
| ARIA ^c^ ** |  |  |  |
| Major cities | 3382 (57.6%) | 1086 (61.4%) | 0.015 |
| Inner regional | 1918 (32.7%) | 534 (30.2%) |  |
| Outer regional / remote | 567 (9.7%) | 148 (8.4%) |  |
| *Injury event* |  |  |  |
| Intent ^d^ |  |  |  |
| Unintentional | 2455 (40.8%) | 892 (48.7%) | <0.001 |
| Intentional self-harm | 3166 (52.6%) | 760 (41.5%) |  |
| Assault | 204 (3.4%) | 108 (5.9%) |  |
| Other / unknown | 194 (3.2%) | 70 (3.8%) |  |
| Trauma type ^e^ |  |  |  |
| Blunt | 2540 (42.3%) | 957 (52.4%) | <0.001 |
| Penetrating | 601 (10.0%) | 103 (5.6%) |  |
| Threat to breathing | 217 (3.6%) | 98 (5.4%) |  |
| Thermal mechanism | 2594 (43.2%) | 658 (36.0%) |  |
| Other | 57 (0.9%) | 11 (0.6%) |  |
| Cause of injury ^f^ |  |  |  |
| Transport Injury Event | 2043 (33.3%) | 743 (39.4%) | <0.001 |
| Low fall (≤1 m) | 23 (0.4%) | 43 (2.3%) |  |
| High fall (>1 m) | 222 (3.6%) | 61 (3.2%) |  |
| Other fall | 85 (1.4%) | 73 (3.9%) |  |
| Hanging | 2111 (34.4%) | 457 (24.2%) |  |
| Other Crushing/Threat to Breathing | 253 (4.1%) | 110 (5.8%) |  |
| Contact with Person | 24 (0.4%) | 8 (0.4%) |  |
| Penetrating injury | 594 (9.7%) | 102 (5.4%) |  |
| Thermal mechanism | 217 (3.5%) | 98 (5.2%) |  |
| Drowning | 282 (4.6%) | 95 (5.0%) |  |
| Other/unknown | 289 (4.7%) | 96 (5.1%) |  |

Missing data: a) n=3 (0.0%); b) n=429 (5.3%); c) n=397 (4.9%); d) n=181 (2.3%); e) n=196 (2.4%); f) n=190 (2.4%)
