# Supplementary material for: Differences in the epidemiology of out-of-hospital and in-hospital trauma deaths
Source: PLoS One. 2019 Jun 4;14(6):e0217158. doi: 10.1371/journal.pone.0217158 (PMC6548370; doi:10.1371/journal.pone.0217158)
Supplement: S1 Fig — (DOCX) [file pone.0217158.s002.docx]

**Sensitivity analysis including Black Saturday bushfire deaths**

The incidence of overall trauma deaths did not change between July 2006 and June 2016 (incidence rate ratio (IRR) = 0.995, 95% confidence interval (CI): 0.989, 1.001; P=0.131). The incidence of out-of-hospital trauma deaths declined 1% per year (IRR = 0.988; 95% CI: 0.980, 0.995; P=0.002) while the incidence of in-hospital trauma deaths increased 1% per year (IRR = 1.013; 95% CI: 1.001, 1.026; P=0.032) (Figure 1).


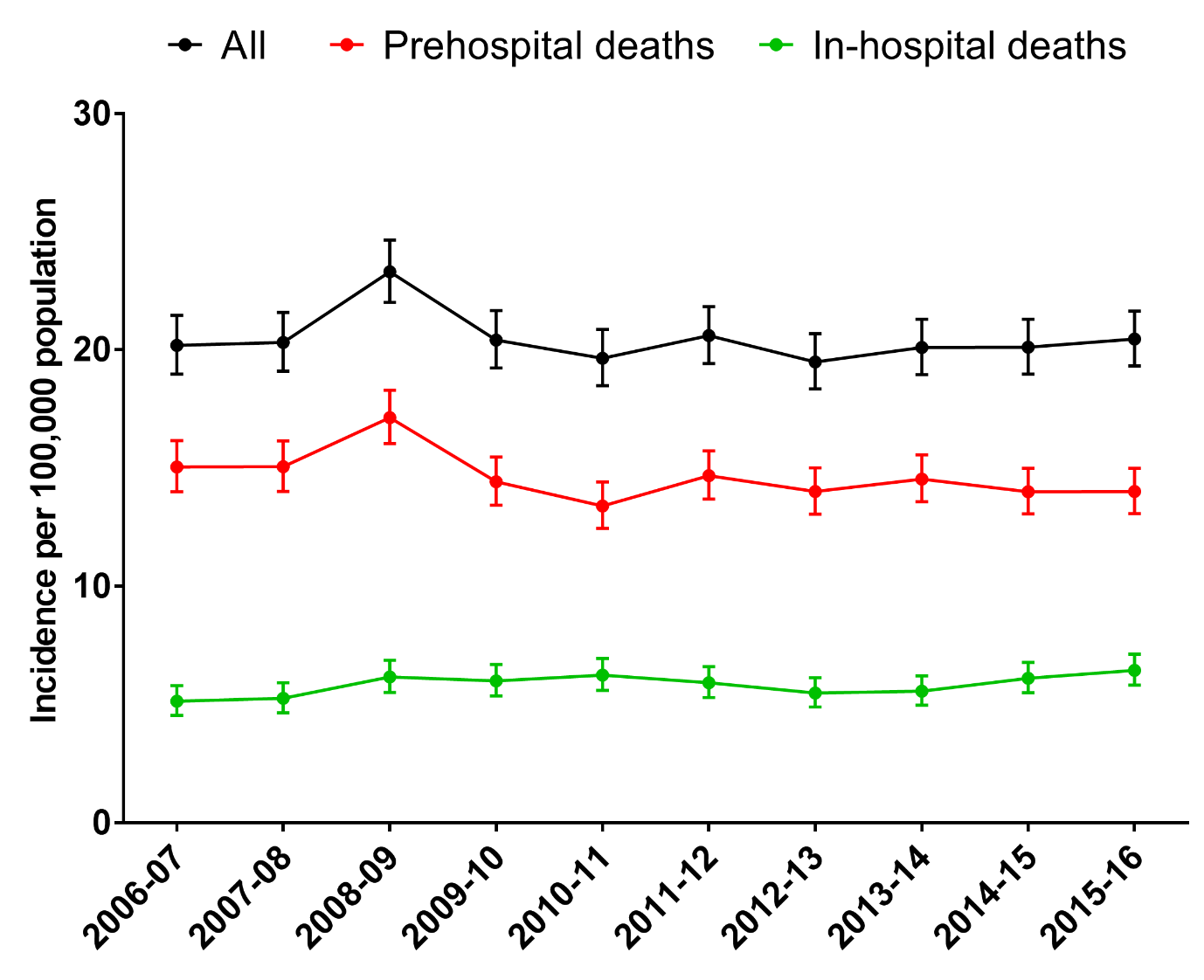


S1 Fig: Incidence of out-of-hospital and in-hospital trauma deaths (including Black Saturday bushfire deaths).
